# Patterns of Adverse Drug Reactions in Different Age Groups: Analysis of Spontaneous Reports by Community Pharmacists

**DOI:** 10.1371/journal.pone.0132916

**Published:** 2015-07-14

**Authors:** Yun Mi Yu, Wan Gyoon Shin, Ju-Yeun Lee, Soo An Choi, Yun Hee Jo, So Jung Youn, Mo Se Lee, Kwang Hoon Choi

**Affiliations:** 1 College of Pharmacy & Research Institute of Pharmaceutical Sciences, Seoul National University, Seoul, South Korea; 2 College of Pharmacy, Institute of Pharmaceutical Science and Technology, Hanyang University, Ansan, South Korea; 3 Regional Pharmacovigilance Center, Korean Pharmaceutical Association, Seoul, South Korea; Dafra Pharma Research and Development, BELGIUM

## Abstract

**Purpose:**

To evaluate the clinical manifestations and causative drugs associated with adverse drug reactions (ADRs) spontaneously reported by community pharmacists and to compare the ADRs by age.

**Methods:**

ADRs reported to the Regional Pharmacovigilance Center of the Korean Pharmaceutical Association by community pharmacists from January 2013 to June 2014 were included. Causality was assessed using the WHO-Uppsala Monitoring Centre system. The patient population was classified into three age groups. We analyzed 31,398 (74.9%) ADRs from 9,705 patients, identified as having a causal relationship, from a total pool of 41,930 ADRs from 9,873 patients. Median patient age was 58.0 years; 66.9% were female.

**Results:**

Gastrointestinal system (34.4%), nervous system (14.4%), and psychiatric (12.1%) disorders were the most frequent symptoms. Prevalent causative drugs were those for acid-related disorders (11.4%), anti-inflammatory products (10.5%), analgesics (7.2%), and antibacterials (7.1%). Comparisons by age revealed diarrhea and antibacterials to be most commonly associated with ADRs in children (p < 0.001), whereas dizziness was prevalent in the elderly (p < 0.001). Anaphylactic reaction was the most frequent serious event (19.7%), mainly associated with cephalosporins and non-steroidal anti-inflammatory drugs. Among 612 ADRs caused by nonprescription drugs, the leading symptoms and causative drugs were skin disorders (29.6%) and non-steroidal anti-inflammatory drugs (16.2%), respectively.

**Conclusions:**

According to the community pharmacist reports, the leading clinical manifestations and causative drugs associated with ADRs in outpatients differed among age groups.

## Introduction

An adverse drug reaction (ADR), as defined by the World Health Organization (WHO), is “a noxious and unintended response of a drug, which occurs at a dose normally used in humans for prophylaxis, diagnosis, or therapy” [1]. Previous reports have suggested that 7–11.2% of ADRs result in hospitalization [2,3] and that the mean cost of ADRs leading to admission was 2721 Euros per patient [4]. Previous studies on ADRs have focused on inpatient care settings. While hospitalized patients are under close medical monitoring, outpatients are not. Because the contact is intermittent and consultation hours are constrained, it is difficult for physicians to secure sufficient communication time to ascertain the presence of ADRs in ambulatory care settings. Thus, the risk and expense of treatment of ADRs in outpatients may increase because remedial action is often delayed [5]. Considering the large proportion of prescriptions issued in ambulatory care, knowledge of ADRs in this population is important to prevent medication-related harm.

In outpatients, community pharmacists (CPs) may effectively monitor patient safety and provide adequate information through medication counseling [6,7]. It is easy for patients to visit community pharmacies because of their wide geographical distribution and accessibility without the need for an appointment. As CPs serve patients with and without prescriptions, their active involvement in ADR monitoring and reporting is likely to improve the scope and quality of spontaneous ADR reporting [8].

In 2013, the Korea Institute of Drug Safety and Risk Management (KIDS) added the regional pharmacovigilance center of the Korean Pharmaceutical Association (RPVC-KPA), to existing RPVCs. While the existing RPVCs targeted each regional hub and their ADR reporting was mainly centered on inpatients in affiliated hospitals [9], the activity of RPVC-KPA was conducted on a national scale and focused on outpatients in community pharmacies nationwide. All CPs can report ADRs to RPVC-KPA through the spontaneous reporting system connected to their pharmacy’s billing program or the KIDS website. Participating community pharmacies comprised 4.0% of the 20,971 registered nationwide community pharmacies in Korea as of March 2014 [10]. The reports by CPs comprised 3.4% of all ADR reports sent to KIDS by healthcare professionals [10]. This is a relatively low proportion in comparison to that in Netherlands, Spain, or Portugal, but it is comparable to the proportion in the UK, France, and Japan [11]. Considering the increase in the proportion of ADR reports by CPs from 0.8% (324 reports) in the first quarter of 2013 to 10.7% (5621 reports) in the second quarter of 2014, the participation of CPs in ADR reporting is expected to expand [10]. Pharmacovigilance in outpatients can be improved by the active participation of CPs.

Although the data from spontaneous ADR reports by CPs may provide more pertinent information for ambulatory patients [12], few studies have been reported on this topic [8]. In addition, few studies have compared the ADR patterns by age group in ambulatory care patients [13]. A systematic review for the ADRs in ambulatory care showed that most studies investigated ADRs leading to hospitalization or emergency department visit [14]. Therefore, we aimed to evaluate the clinical manifestations and causative drugs associated with ADRs spontaneously reported by CPs and compare the ADRs by age.

## Materials and Methods

ADRs spontaneously reported to RPC-KPA by CPs nationwide from January 2013 to June 2014 were collected. According to the WHO definition, this study only included ADRs associated with a dose normally used in humans and reports associated with a drug administered for ordinary prophylactic or therapeutic purposes. Reports related to drug abuse, suicide attempts, or medication errors were excluded. To reduce the possibility of duplication, each ADR was individually compared based on the patient’s age, sex, and residence; location of the participating pharmacy; date of onset of the reaction; and related drugs.

The patient population was classified into three age groups: children (less than 18 years), adult (19–63 years), and elderly (more than 64 years) groups. Reports without age were excluded. Patient records were anonymized and de-identified prior to analysis. The Institutional Review Board of Seoul National University approved this study (IRB No. 1405/002-006) and waived the requirement of informed consent.

The causality of a drug for ADR was assessed using the World Health Organization-Uppsala Monitoring Centre (WHO-UMC) criteria, which was composed of six categories: certain, probable, possible, unlikely, conditional, and unassessable [15]. Causality was independently assessed by two trained pharmacists. When the pharmacists disagreed on causality, they discussed the difference and achieved consensus in all cases. The inter-rater reliability in initial assessment was calculated and Cohen’s κ score greater than 0.81 was considered “very good agreement” [16]. ADRs classified as “less than possible” in the causality assessment were excluded from subsequent analysis.

Clinical manifestations were classified using the WHO-adverse reaction terminology (ART) system [17]. The system-organ classes (SOC) and the preferred terms (PT) of the WHO-ART system were used as a main- and sub-category, respectively. Symptoms matched with the same PT were treated as the same event. Two or more PTs reported in one patient and two or more medications involved in one event were counted as different ADRs. The causative drugs were classified using the Anatomical Therapeutic Chemical (ATC) classification system [18].

The frequency of clinical manifestations and causative drugs was compared according to age group. Unlabeled ADRs were identified by assessing whether reported ADRs were included in the label of each causative drug. The relationship between serious ADRs and causative drugs was evaluated by comparing the count of specific ADRs according to specific drugs. Serious ADRs were defined as cases that were fatal, caused hospitalization or persistent disability, or were life-threatening according to WHO criteria [19]. The patterns of ADRs caused by nonprescription drugs were also analyzed by comparing the number of specific clinical manifestations according to specific drugs.

### Statistics

Descriptive statistics were used to summarize the demographic and clinical characteristics of study participants. Means and standard deviations were used for continuous variables, whereas frequencies and percentages were used for categorical variables. The categorical characteristics of three age groups including children, adults, and elderly were compared. Chi-squared test or Fisher’s exact test was applied to compare categorical variables between groups. The significance level was set at p < 0.01. For post hoc analysis, chi-squared test or Fisher’s exact test with Bonferroni correction was employed and the significance level was set at p < 0.003. Data analysis was performed using SPSS version 21.0 (SPSS Inc., Chicago, IL).

## Results

From January 2013 to June 2014, 42,018 ADRs from 9,919 patients were reported. A total of 920 community pharmacies participated. The proportion of participating community pharmacies located in metropolitan versus rural areas was 59.4% versus 40.6%. Forty-six patients (88 ADRs) were excluded because of a lack of information about age. Causality assessment using WHO-UMC criteria for 41,930 ADRs in 9,873 patients classified 1.4% as certain, 5.4% as probable, 68.1% as possible, 24.7% as unlikely, 0.2% as conditional, and 0.2% as unassessable. The κ score was 0.83 showing "very good agreement" between the initial assessments of causality. After exclusion of the 10,532 ADRs (25.1%) having a less than possible degree of causality, 31,398 ADRs (74.9%) in 9,705 patients were analyzed. The mean number of events per patient was 1.4 and the mean number of causative drugs per event was 2.3.

### Demographic Characteristics

The median age of the 9,705 patients was 58.0 years, ranging 3 months to 98 years (Table 1). The adult group comprised the largest portion of patients (64.0%), followed by the elderly group (32.5%) and children (3.5%). Females comprised 66.9% of all patients, with similar distributions in the adult and elderly subgroups. In contrast, female children comprised less than half of the pediatric group, which represented a significant difference from the other age groups (p < 0.001).

**Table 1:** Patient demographics.

Characteristics	Value
Number of patients	9,705
Female (%)	66.9
Age, median (range, years)	58.0 (0.3–98.0)
Age, n (%)	
**Children**	**341 (3.5)**
<2 years	61 (0.6)
2–11 years	165 (1.7)
12–18 years	115 (1.2)
**Adults (19–64 years)**	**6,209 (64.0)**
**Elderly**	**3,155 (32.5)**
65–74 years	2,076 (21.4)
75–84 years	965 (9.9)
≥85 years	114 (1.2)
Reported events per patient (mean)	1.4
Reported drugs per event (mean)	2.3
Number of patients with serious events, n	52

### Clinical Manifestations of Adverse Drug Reactions

The clinical manifestations most frequently associated with ADRs were gastro-intestinal (GI) system disorders (4,623 events, 34.4%) followed by nervous system disorders (1,932 events, 14.4%) and psychiatric disorders (1,620 events, 12.1%). The most common symptoms were dizziness (1,142 events, 8.5%), dyspepsia (1,139 events, 8.5%), and somnolence (847 events, 6.3%).

A comparison of clinical manifestations according to age revealed that GI system disorders and diarrhea were most common in children, but dry mouth was least frequent in this group (p < 0.001) (S1 Table). The leading drugs causing diarrhea in children were antibacterial agents. The elderly group showed a significantly higher frequency of ADRs involved in nervous and urinary system disorders (p < 0.001). Dizziness was reported more frequently in the elderly than in any other age group (p < 0.001). The main drugs causing dizziness in elderly were analgesics and antiepileptics. Psychiatric disorders (including their subcategory somnolence) and skin disorders (including their subgroup rash and urticarial) were more frequent in children and adults (p < 0.001) (S1 Table).

### Causative Drugs

The most prevalent causative drugs were alimentary tract and metabolism drugs (6,984 ADRs, 22.2%), followed by musculoskeletal system drugs (5,436 ADRs, 17.3%) and nervous system drugs (5,210 ADRs, 16.6%). According to the subclassification, drugs for acid-related disorders (3,588 ADRs, 11.4%), anti-inflammatory products (3,305 ADRs, 10.5%), analgesics (2,262 ADRs, 7.2%), and antibacterials (2,240 ADRs, 7.1%) were frequently associated with ADRs.

**Fig 1:**
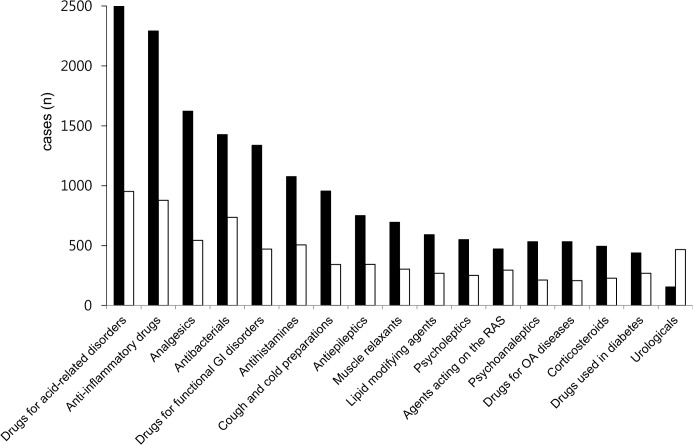
Frequency of adverse drug reactions and causative drugs according to sex. GI, gastrointestinal; RAS, renin-angiotensin system; OA, obstructive airway. Black bars: female; white bars: male.

Drugs acting on the respiratory system and anti-infective drugs were more frequently involved in ADRs in the pediatric population than in other groups (p < 0.001). Drugs for the nervous system, cardiovascular system, genitourinary system and sex hormones, and blood and blood-forming organs were reported more frequently as causative drugs for ADRs in the elderly (p < 0.001) (S2 Table). Unlabeled ADRs were not identified. A comparison of causative drugs according to sex revealed that urological agents were more prevalently involved in ADRs in males (p < 0.001) (Fig 1).

### Serious Events

In total, 66 serious events were identified in 52 patients who experienced a life-threatening event (15 patients), hospitalization (36 patients), or persistent disability (1 patient). The life-threatening events included symptoms associated with anaphylactic reactions, dyspnea, and circulatory failure. The persistent disability involved blindness and ocular hemorrhage associated with everolimus, an antineoplastic agent. The proportion of serious events in adults and elderly groups was 0.58% and 0.51%, respectively. There were no serious event reports for the pediatric population. Among serious events, the most common symptoms were anaphylactic reaction (13 events, 19.7%). Cephalosporins and non-steroidal anti-inflammatory drugs (NSAIDs) were most frequently associated with this symptom. Non-steroidal anti-inflammatory drugs (NSAIDs) (18 ADRs, 19.8%), analgesics (17 ADRs, 18.7%), and antibacterials (13 ADRs, 14.3%) were the main causative agents for serious adverse events (Table 2).

**Table 2:** Causative drugs and clinical manifestation in serious events.

Causative drugs	Number of ADRs (%)	Clinical manifestation (n)^a^
Anti-inflammatory products^b^	18 (19.8)	AR (8), edema (2), vomiting (2), dizziness (2), CF, uterine hemorrhage, bullous eruption, vision abnormal
Analgesics^c^	17 (18.7)	AR (3), vomiting (5), AP, dizziness (5), headache, HE increased, dyspnea
Antibacterials^d^	13 (14.3)	AR (9), AP, GI hemorrhage (2), dyspepsia
Urologicals	5 (5.5)	asthenia, dizziness, hypotension postural, dysuria (2)
Psychoanaleptics	4 (4.4)	vomiting, AP, dysuria, palpitation
Drugs used in diabetes	4 (4.4)	CF, dyspnea, hypoglycemia (2)
Antithrombotic agents	4 (4.4)	GI hemorrhage (2), CF, dyspnea
Antiepileptics	2 (2.2)	vomiting, dizziness
Digestives	2 (2.2)	tongue disorder, dyskinesia
Drugs for acid related disorders	2 (2.2)	GI hemorrhage, hypertension
Drugs for functional GI disorders	2 (2.2)	vocal cord paralysis, dystonia
Sex hormones	2 (2.2)	dizziness, intermenstrual bleeding
Cough and cold preparations	2 (2.2)	AR, rash
Drugs for OA disease	2 (2.2)	AR, AP
Peripheral vasodilators	2 (2.2)	vomiting, dizziness
Antineoplastic agents	2 (2.2)	blindness, ocular hemorrhage
Antivirals	1 (1.1)	vomiting
Antimycotics	1 (1.1)	HE increased
Nasal preparations	1 (1.1)	dysuria
Cardiac therapy	1 (1.1)	CF
Lipid modifying agents	1 (1.1)	CF
Agents acting on the RAS	1 (1.1)	vomiting
Immunosuppressants	1 (1.1)	HE increased
Corticosteroids	1 (1.1)	HE increased

ADRs, adverse drug reactions; GI, gastrointestinal; OA, obstructive airway diseases; RAS, renin-angiotensin system; AR, anaphylactic reaction; AP, abdominal pain; HE, hepatic enzyme; CF, circulatory failure.

^a^Numbers in parentheses indicate the number of adverse drug reactions.

^b^Dexibuprofen, loxoprofen, and celecoxib, etc.

^c^Combination of acetaminophen and tramadol, buprenorphine, and sumatriptan, etc.

^d^Cefaclor, cefadroxil, and amoxicillin, etc.

### Nonprescription Drugs

Nonprescription drugs were implicated in 394 patients and 680 ADRs. The adult group comprised the largest portion of patients (76.4%), followed by the elderly group (18.8%) and children (4.8%). Skin disorders (181 events, 29.6%) including rash and pruritus were the most frequently reported manifestations, followed by GI system disorders (155 events, 25.3%) such as dyspepsia and nausea. Among a total of 186 causative drugs, NSAIDs (110 ADRs, 16.2%) and topical products for joint and muscular pain (56 ADRs, 8.2%) were most common. A combination drug containing acetaminophen and chlorzoxazone (40 ADRs, 5.8%) was the most prevalent individual drug, followed by naproxen (37 ADRs, 5.4%) and ibuprofen (29 ADRs, 4.2%) (Table 3). A comparison of ADRs by nonprescription drugs according to age in 394 patients revealed that NSAIDs and GI system disorders were more frequently involved in children than in other groups (p < 0.001). NSAIDs and GI system disorders respectively comprised 48.7% of the ADRs by nonprescription drugs in the pediatric group.

**Table 3:** Causative drugs and clinical manifestation among the nonprescription drugs.^a^

Causative drugs	Number of ADRs (%)	Clinical manifestation (n)^b^
**Musculo-skeletal system**		
Acetaminophen/chlorzoxazone	40 (5.8)	dizziness (10), pruritus (3), urticaria (3), dyspepsia (3), vomiting (3), nausea (3), rash (2)
Naproxen	37 (5.4)	dyspepsia (6), edema (4), pruritus (4), rash (4), abdominal pain (2)
Ibuprofen	29 (4.2)	dyspepsia (4), vomiting (3), abdominal pain (3), urticaria (3), dizziness (2)
Ketoprofen patch	17 (2.5)	rash (6), pruritus (4), skin exfoliation (2)
Dexibuprofen	16 (2.3)	urticaria (6), edema periorbital (3), dizziness (2)
Ibuprofen arginine	15 (2.2)	edema periorbital (4), pruritus (3), nausea (2), sweating increase (2)
Flurbiprofen patch	13 (1.9)	rash (4), pruritus (2), skin exfoliation (2), dermatitis (2)
Clonixin	9 (1.3)	urticaria (3)
**Alimentary tract**		
Antacid combinations^c^	10 (1.4)	constipation (2)
**Respiratory system**		
Pseudoephedrine/triprolidine	18 (2.6)	insomnia (4), dizziness (2), sweating increase (2), somnolence (2)
Cetirizine	11 (1.6)	headache (2), somnolence (2)
Flurbiprofen 8.75 mg	9 (1.3)	dizziness (2), palpitation (2)
**Nervous system**		
Acetaminophen/methionine	25 (3.6)	edema (3), nausea (3), rash (3), urticaria (2), vomiting (2), dyspnea (2), drug dependence (2)
Gingko leaf ext.	10 (1.4)	pruritus (2), dyspepsia (2)
Nicotine patch	9 (1.3)	dermatitis (3), pruritus (2)
Diphenhydramine	7 (1.0)	abdominal pain (2)
**GU system and sex hormones**		
Agnus Castus fruit ext.	18 (2.6)	abdominal pain (4), rash (2), nausea (2), acne (2)
Desogestrel/ethinyl Estradiol	14 (2.0)	menstrual disorder (4), nausea (2), weight increase (2)
Gestodene/ethinyl Estradiol	12 (1.7)	edema (2), acne (2)
**Dermatologicals**		
Benzoyl peroxide ointment	8 (1.2)	rash (3), pruritus (2)

ADRs, adverse drug reactions; GU, genito-urinary.

^a^680 adverse drug reactions from 394 patients.

^b^Clinical manifestations reported for more than one adverse drug reaction and the number of adverse drug reactions.

^c^Combinations of aluminum magnesium silicate/ranitidine/magnesium oxide/aluminum magnesium hydroxide.

## Discussion

To the best of our knowledge, this is the first large-scale study of CP-reported ADRs in outpatients in Korea. Reports of clinical manifestations affecting the GI system, nervous system, and psychiatric disorders were prevalent. The most frequent causative drugs were those used to treat acid-related disorders, anti-inflammatory products, analgesics, and antibacterials. ADR patterns differed by age group. Our findings suggest the need to establish pharmacovigilance strategies adapted to outpatient characteristics and age group.

In this study, females comprised around two-thirds (66.9%) of the study cohort who had experienced ADRs, which could be explained by the epidemiological population distribution (female, 58.3%) among the average daily number of outpatients [20]. A multinational study reported that the ADR reporting rate of antidepressants was not significantly different between men and women when considering drug consumption [21]. However, other studies have suggested a preponderance of ADRs in female patients [13,22,23]. The higher adverse event rate in females has been found to result from differences in pharmacokinetic factors [22], hormonal factors [24], drug prescription rate [23], medical care utilization [20,25], propensity of symptom reporting [25], and a historical lack of drug research in this population [26]. In the present study, women also experienced more than twice the number of anaphylactic reactions compared to men. Ribeiro-Vaz et al. also showed that females are more likely to experience anaphylaxis [27].

Comparison of the ADR reports by CPs with the entire set of ADR reports to KIDS during the same period showed that the prevalent ADR symptoms were GI and nervous system disorders and the most frequent causative drugs were anti-inflammatory products, analgesics, and antibacterials in both reports [10]. However, the proportion of serious events (0.54%) in reports by CPs was much lower than that in the entire ADR dataset (11.2%) [10], which can be explained mainly by the relatively less severe medical state of outpatients and by the limited experience of CPs in ADR reporting. The non-seriousness that prevails in early periods of pharmacovigilance by a new expert group may be one of the reasons for the low proportion of serious events in this study [28].

The clinical manifestations and causative drugs showed specific trends according to age. In the pediatric group, GI system disorders, especially diarrhea, and antibacterial agents were most frequent. These results are consistent with previous reports. Two systematic reviews and a prospective cohort study showed that antibacterial agents and GI disorders were the leading causes and symptoms, respectively, of ADRs in pediatric outpatients [14,29,30]. In this study, antibacterial agents comprised 46.1% of the drugs causing diarrhea in children. Infants aged less than 24 months and patients taking broad-spectrum penicillins or cephalosporins accounted for 37.0% and 81.9% of the children who experienced antibacterial-associated diarrhea. These results are consistent with the risk associated with reduced fecal flora in infants and broad-spectrum penicillins and cephalosporins in pediatric diarrhea [31,32].

Dizziness was the most common symptom in the elderly, consistent with reports of a 30% prevalence in older populations [33]. Maarsingh et al. showed that medications were the second leading cause of dizziness following comorbidities such as cardiovascular and peripheral vestibular disease in the elderly [34]. In this study, the main drugs associated with dizziness were those used to treat the nervous system (29.5%), such as combination drugs containing acetaminophen and tramadol (9.3%), gabapentin (3.9%), and pregabalin (3.8%). Considering the risk of secondary injury resulting from dizziness in the elderly, use of these drugs should be carefully monitored and evaluated.

Cephalosporin antibiotics and NSAIDs were mainly associated with anaphylactic reactions, which was the major clinical manifestation in serious events. A review of a decade of spontaneous ADR reports showed similar results; antibiotics and the combination of NSAIDs and acetaminophen were primarily responsible for the incidence of anaphylaxis [27].

For nonprescription drugs, skin and GI system disorders were most prevalent, and were chiefly caused by NSAIDs such as naproxen and ibuprofen. A prospective multi-center study also reported that the most frequent nonprescription drugs causing ADR-related hospital admissions were NSAIDs including aspirin, diclofenac, and ibuprofen; the leading symptoms were GI disorders [35].

In summary, among the outpatient ADRs spontaneously reported by CPs, those involving the GI system, nervous system, and psychiatric disorders were prevalent. Anti-inflammatory products, analgesics, and antibacterials were the leading causes of ADRs, including serious events. The patterns of outpatient ADRs reported by CPs also differed between age groups.

This study has several limitations. First, we relied on spontaneous reporting, which is subject to under-reporting and lack of information [36,37]. All data were retrospective and we were unable to confirm accuracy or replace missing data. However, spontaneous reporting by CPs has the advantages of providing the direct outpatient complaints [38]. Second, although these pharmacovigilance systems are intended to detect signals, unlabeled ADRs were not identified; therefore, we could not suggest any potential signals. Third, we could not account for the size of the at-risk population because of a lack of information on substantial drug usage (the number of prescriptions for each causative drug at each participating pharmacy) in outpatients. Because commonly prescribed drugs are more likely to be the offenders in ADR events [39], considering the prevalence of drug usage might aid in the interpretation of ADR frequency.

## Supporting Information

S1 Table Clinical manifestation of adverse drug reactions according to the system-organ classification and preferred terms. (DOCX)

S2 Table Causative drugs for adverse drug reactions according to the anatomical therapeutic chemical classification system. (DOCX)
