# Supplementary material for: Patterns of Adverse Drug Reactions in Different Age Groups: Analysis of Spontaneous Reports by Community Pharmacists
Source: PLoS One. 2015 Jul 14;10(7):e0132916. doi: 10.1371/journal.pone.0132916 (PMC4501755; doi:10.1371/journal.pone.0132916)
Supplement: S1 Table — (DOCX) [file pone.0132916.s001.docx]

**Text S1. Patterns of Adverse Drug Reactions in Different Age Groups: Analysis of Spontaneous Reports by Community Pharmacists**

**S1 Table.** Clinical manifestation of adverse drug reactions according to the system-organ classification and preferred terms.^d^

| **Clinical manifestation** | **Total (%)** | **Children^a^** | **Adults^b^** | **Elderly^c^** | **p value^e^** | **Post hoc^f^** |
| --- | --- | --- | --- | --- | --- | --- |
| **Gastro-intestinal system disorders** | **4,623 (34.4)** | **200 (46.3)** | **2,875 (33.3)** | **1,548 (35.4)** | **<0.001** | **a>b,c** |
| Dyspepsia | 1,139 (8.5) | 4 (0.9) | 747 (8.7) | 388 (8.9) | <0.001 | a<b,c |
| Nausea | 802 (6.0) | 20 (4.6) | 550 (6.4) | 232 (5.3) | 0.025 |  |
| Diarrhea | 575 (4.3) | 115 (26.6) | 311 (3.6) | 149 (3.4) | <0.001 | a>b,c |
| Dry mouth | 570 (4.2) | 2 (0.5) | 315 (3.7) | 253 (5.8) | <0.001 | a<b<c |
| Vomiting | 483 (3.6) | 23 (5.3) | 291 (3.4) | 169 (3.9) | 0.053 |  |
| Constipation | 401 (3.0) | 10 (2.3) | 249 (2.9) | 142 (3.2) | 0.371 |  |
| Abdominal pain | 324 (2.4) | 16 (3.7) | 196 (2.3) | 112 (2.6) | 0.123 |  |
| **Nervous system**  **disorders** | **1,932 (14.4)** | **25 (5.8)** | **1,160 (13.4)** | **747 (17.1)** | **<0.001** | **c>b>a** |
| Dizziness | 1,142 (8.5) | 5 (1.2) | 658 (7.6) | 479 (10.9) | <0.001 | c>b>a |
| Headache | 366 (2.7) | 3 (0.7) | 243 (2.8) | 120 (2.7) | 0.03 |  |
| **Psychiatric disorders** | **1,620 (12.1)** | **71 (16.4)** | **1,111 (12.9)** | **438 (10.0)** | **<0.001** | **c<a,b** |
| Somnolence | 847 (6.3) | 37 (8.6) | 635 (7.4) | 175 (4.0) | <0.001 | c<a,b |
| Insomnia | 446 (3.3) | 15 (3.5) | 279 (3.2) | 152 (3.5) | 0.758 |  |
| **General disorders** | **1,551 (11.5)** | **38 (8.8)** | **994 (11.5)** | **519 (11.9)** | **0.163** |  |
| Edema | 380 (2.8) | 3 (0.7) | 254 (2.9) | 123 (2.8) | 0.023 |  |
| Asthenia | 314 (2.3) | 9 (2.1) | 204 (2.4) | 101 (2.3) | 0.92 |  |
| **Skin disorders** | **1,543 (11.5)** | **68 (15.7)** | **1,095 (12.7)** | **380 (8.7)** | **<0.001** | **c<a,b** |
| Pruritus | 457 (3.4) | 15 (3.5) | 289 (3.3) | 153 (3.5) | 0.906 |  |
| Rash | 401 (3.0) | 27 (6.3) | 294 (3.4) | 80 (1.8) | <0.001 | c<a,b |
| Urticaria | 191 (1.4) | 11 (2.5) | 150 (1.7) | 30 (0.7) | <0.001 | c<a,b |
| **Urinary system**  **disorders** | **572 (4.3)** | **8 (1.9)** | **328 (3.8)** | **236 (5.4)** | **<0.001** | **c>a,b** |
| Face edema | 340 (2.5) | 6 (1.4) | 214 (2.5) | 120 (2.7) | 0.205 |  |
| **Cardiovascular disorders** | **320 (2.4)** | **4 (0.9)** | **210 (2.4)** | **106 (2.4)** | **0.131** |  |
| Palpitation | 156 (1.2) | 2 (0.5) | 115 (1.3) | 39 (0.9) | 0.033 |  |
| **Respiratory system**  **disorders** | **252 (1.9)** | **3 (0.7)** | **166 (1.9)** | **83 (1.9)** | **0.183** |  |
| **Metabolic disorders** | **227 (1.7)** | **3 (0.7)** | **161 (1.9)** | **63 (1.4)** | **0.054** |  |
| **Musculo-skeletal system disorders** | **211 (1.6)** | **1 (0.2)** | **129 (1.5)** | **81 (1.9)** | **0.023** |  |

^d^Number of events and percentage of individual events within each group.

^e^Chi-squared test of the three groups.

^f^ Bonferroni correction (p < 0.003) with chi-squared or Fisher’s exact test.
