# Supplementary material for: Patterns of Adverse Drug Reactions in Different Age Groups: Analysis of Spontaneous Reports by Community Pharmacists
Source: PLoS One. 2015 Jul 14;10(7):e0132916. doi: 10.1371/journal.pone.0132916 (PMC4501755; doi:10.1371/journal.pone.0132916)
Supplement: S2 Table — (DOCX) [file pone.0132916.s002.docx]

**Text S1. Patterns of Adverse Drug Reactions in Different Age Groups: Analysis of Spontaneous Reports by Community Pharmacists**

**S2 Table.** Causative drugs for adverse drug reactions according to the anatomical therapeutic chemical classification system.^d^

| **Causative drugs** | **Total (%)** | **Children^a^** | **Adults^b^** | **Elderly^c^** | **p value^e^** | **Post hoc^g^** |
| --- | --- | --- | --- | --- | --- | --- |
| **Alimentary tract and metabolism^h^** | **6,984 (22.2)** | **74 (7.3)** | **4,564 (22.8)** | **2,346 (22.6)** | **<0.001** | **a<b,c** |
| Drugs for acid related disorders | 3,588 (11.4) | 30 (3.0) | 2,359 (11.8) | 1,199 (11.6) | <0.001 | a<b,c |
| Drugs for functional GI disorders | 1,850 (5.9) | 22 (2.2) | 1,232 (6.2) | 596 (5.8) | <0.001 | a<b,c |
| Drugs used in diabetes | 713 (2.3) | 0 (0) | 430 (2.1) | 283 (2.7) | <0.001^f^ |  |
| Others | 833 (2.7) | 22 (2.2) | 543 (2.7) | 268 (2.6) |  |  |
| **Musculo-skeletal system^i^** | **5,436 (17.3)** | **130 (12.9)** | **3,630 (18.1)** | **1,676 (16.2)** | **<0.001** | **b>a,c** |
| Anti-inflammatory products | 3,305 (10.5) | 100 (9.9) | 2,233 (11.2) | 972 (9.4) | <0.001 | c<b |
| Muscle relaxants | 1,057 (3.4) | 2 (0.2) | 721 (3.6) | 334 (3.2) | <0.001 | a<b,c |
| Others | 1,074 (3.4) | 28 (2.8) | 676 (3.4) | 370 (3.6) |  |  |
| **Nervous system^j^** | **5,210 (16.6)** | **82 (8.1)** | **3,040 (15.2)** | **2,088 (20.1)** | **<0.001** | **a<b<c** |
| Analgesics | 2,262 (7.2) | 33 (3.3) | 1,444 (7.2) | 785 (7.6) | <0.001 | a<b,c |
| Antiepileptics | 1,131 (3.6) | 26 (2.6) | 649 (3.2) | 456 (4.4) | <0.001 | b<c |
| Psycholeptics | 812 (2.6) | 8 (0.8) | 443 (2.2) | 361 (3.5) | <0.001 | c>a,b |
| Psychoanaleptics | 774 (2.5) | 13 (1.3) | 383 (1.9) | 378 (3.6) | <0.001 | c>a,b |
| Others | 231 (0.7) | 2 (0.2) | 121 (0.6) | 108 (1.0) |  |  |
| **Respiratory system^k^** | **4,280 (13.6)** | **389 (38.5)** | **2,784 (13.9)** | **1,107 (10.7)** | **<0.001** | **a>b>c** |
| Antihistamines | 1,589 (5.1) | 128 (12.7) | 1,079 (5.4) | 391 (3.8) | <0.001 | a>b>c |
| Cough/cold preparations | 1,343 (4.3) | 118 (11.7) | 874 (4.4) | 351 (3.4) | <0.001 | a>b>c |
| Drugs for OA diseases | 769 (2.4) | 80 (7.9) | 433 (2.2) | 256 (2.5) | <0.001 | a>b,c |
| Nasal preparations | 491 (1.6) | 51 (5.0) | 347 (1.7) | 93 (0.9) | <0.001 | a>b>c |
| others | 79 (0.3) | 12 (1.2) | 51 (0.3) | 16 (0.2) |  |  |
| **Cardiovascular system^l^** | **3,207 (10.2)** | **4 (0.4)** | **1,794 (9.0)** | **1,409 (13.6)** | **<0.001** | **a<b<c** |
| Lipid modifying agents | 875 (2.8) | 1 (0.1) | 536 (2.7) | 338 (3.3) | <0.001 | a<b,c |
| Agents acting on the RAS | 783 (2.5) | 1 (0.1) | 442 (2.2) | 340 (3.3) | <0.001 | a<b<c |
| Calcium channel blockers | 375 (1.2) | 0 (0) | 214 (1.1) | 161 (1.6) | <0.001^f^ |  |
| Others | 1,174 (3.7) | 2 (0.2) | 602 (3.0) | 570 (5.5) |  |  |
| **Antiinfectives for systemic use^m^** | **2,658 (8.5)** | **262 (25.9)** | **1,880 (9.4)** | **516 (5.0)** | **<0.001** | **a>b>c** |
| Antibacterials | 2,240 (7.1) | 252 (25.0) | 1.584 (7.9) | 404 (3.9) | <0.001 | a>b>c |
| Others | 418 (1.3) | 10 (1.0) | 296 (1.5) | 112 (1.1) |  |  |
| **GU system and sex hormones^n^** | **874 (2.8)** | **2 (0.2)** | **495 (2.5)** | **377 (3.6)** | **<0.001** | **a<b<c** |
| Urologicals | 629 (2.0) | 1 (0.1) | 278 (1.4) | 350 (3.4) | <0.001 | a<b<c |
| Others | 245 (0.8) | 1 (0.1) | 217 (1.1) | 27 (0.3) |  |  |
| **Systemic hormonal preparations^o^** | **848 (2.7)** | **34 (3.4)** | **611 (3.1)** | **203 (2.0)** | **<0.001** | **c<a,b** |
| Corticosteroids | 733 (2.3) | 33 (3.3) | 536 (2.7) | 164 (1.6) | <0.001 | c<a,b |
| Others | 115 (0.4) | 1 (0.1) | 75 (0.4) | 39 (0.4) |  |  |
| **Blood and blood forming organs^p^** | **768 (2.4)** | **3 (0.3)** | **412 (2.1)** | **353 (3.4)** | **<0.001** | **a<b<c** |
| Antithrombotic agents | 597 (1.9) | 1 (0.1) | 292 (1.5) | 304 (2.9) | <0.001 | a<b<c |
| Others | 171 (0.5) | 2 (0.2) | 120 (0.6) | 49 (0.5) |  |  |
| **Antineoplastics^q^** | **546 (1.7)** | **7 (0.7)** | **434 (2.2)** | **105 (1.0)** | **<0.001** | **b>a,c** |
| **Dermatologicals^r^** | **252 (0.8)** | **14 (1.4)** | **185 (0.9)** | **53 (0.5)** | **<0.001** | **c<a,b** |
| **Sensory organs^s^** | **250 (0.8)** | **6 (0.6)** | **133 (0.7)** | **111 (1.1)** | **0.001** | **c>b** |
| **Others** | **85 (0.3)** | **3 (0.3)** | **61 (0.3)** | **21 (0.2)** |  |  |

GI, gastro-intestinal; OA, obstructive airway; GU, genito-urinary.

^d^Number of adverse drug reactions (ADRs) and percentage of individual ADRs within each group.

^e^Chi-squared test among the three groups.

^f^Chi-squared test between the adult and elderly groups.

^g^Bonferroni correction (p < 0.003) with chi-squared test or Fisher’s exact test.

^h^Ranitidine, pantoprazole, levosulpiride, trimbutine, metformin, and glimepiride, etc.

^i^Ibuprofen, loxoprofen, chlorphenesin, and orphenadrine, etc.

^j^Acetaminophen, combination of acetaminophen and tramadol, valproate, gabapentin, alprazolam, risperidone, donepezil, and duloxetine, etc.

^k^Olopatadine, levocetirizine, combination of chlorpheniramine and dihydrocodeine, combination of acetaminophen and ephedrine, montelukast, theophylline, combination of pseudoephedrine and triprolidine, and pseudoephedrine, etc.

^l^Atorvastatin, pravastatin, combination of valsartan and amlodipine, combination of telmisartan and amlodipine, amlodipine, and diltiazem, etc.

^m^Combination of amoxicillin and clavulanate, cefaclor, and ciprofloxacin, etc.

^n^Pentosan polysulfate, terazosin, and tolterodine, etc.

^o^Dexamethasone, triamcinolone, and methylprednisolone, etc.

^p^Aspirin, clopidogrel, and warfarin, etc.

^q^Cyclosporine, mycophenolate, and capecitabine, etc.

^r^Terbinafine, clobetasol propionate, and benzoyl Peroxide, etc.

^s^Hyaluronate, timolol, and fluorometholone, etc.
